# Stopping-power ratio of mouthpiece materials for charged-particle therapy in head and neck cancer

**DOI:** 10.1007/s12194-021-00643-1

**Published:** 2021-11-25

**Authors:** Hiroaki Ikawa, Taku Inaniwa, Masashi Koto, Tapesh Bhattacharyya, Takashi Kaneko, Hirotoshi Takiyama, Makoto Shinoto, Shigeru Yamada, Hiroshi Tsuji

**Affiliations:** 1 QST Hospital, National Institutes for Quantum Science and Technology, 4-9-1 Anagawa, Inage-ku, Chiba, 263-8555 Japan; 2 Department of Accelerator and Medical Physics, National Institute of Radiological Sciences, National Institutes for Quantum Science and Technology, Chiba, Japan

**Keywords:** Particle therapy, Carbon-ion radiation therapy, Proton beam therapy, Custom made mouthpiece, Bite block, Intraoral stent

## Abstract

In this study, the stopping-power ratios (SPRs) of mouthpiece materials were measured and the errors in the predicted SPRs based on conversion table values were further investigated. The SPRs of the five mouthpiece materials were predicted from their computed tomography (CT) numbers using a calibrated conversion table. Independently, the SPRs of the materials were measured from the Bragg peak shift of a carbon-ion beam passing through the materials. The errors in the SPRs of the materials were determined as the difference between the predicted and measured values. The measured SPRs (errors) of the Nipoflex 710™ and Bioplast™ ethylene–vinyl acetate copolymers (EVAs) were 0.997 (0.023) and 0.982 (0.007), respectively. The SPRs of the vinyl silicon impression material, light-curable resin, and bis-acrylic resin were 1.517 (0.134), 1.161 (0.068), and 1.26 (0.101), respectively. Among the five tested materials, the EVAs had the lowest SPR errors, indicating the highest human-tissue equivalency.

## Introduction

In external beam radiation therapy (RT) for head and neck cancer, a mouthpiece is often used to improve positional accuracy and obtain better reproducibility [1, 2]. Doi et al. [1] reported that the use of a mouthpiece in photon-based RT for head and neck cancer patients resulted in a significant reduction in setup errors. The mouthpiece should effectively reduce setup errors in charged-particle head and neck cancer treatments. Moreover, the mouthpiece should reduce the side effects of external beam RT [3–10]. Verrone et al. [7] reported that the use of a mouthpiece during intensity-modulated RT for oral cancer effectively reduced the dose delivered to the healthy tissues such as the maxilla and parotid gland. The reduced side effects of custom-made mouthpieces have also been reported for proton [11] and carbon-ion (C-ion) RT [3, 4, 12].

Particle beams have unique physical characteristics that are distinct from those of photons. The superiority of particle beams is attributed to their low entrance dose, finite range, and an extremely steep increase in the dose within the beam’s range, known as the Bragg peak, which enables improved target coverage and better sparing of organs at risk (OARs) in close proximity to the target [13, 14]. However, the range in a patient is associated with considerable uncertainty due to imaging, patient setup, beam delivery, and dose calculation. This error range may cause an underdose to the target and an overdose to the OARs, leading to decreased local control and unexpected side effects. Hence, beam range accuracy is of utmost importance and requires accurate calculation during treatment planning to utilize its full potential.

In charged-particle therapy, computed tomography (CT) images are used for treatment planning and patient dose calculations. For CT-based planning, the CT value is converted to the stopping-power ratio (SPR) of the tissues with respect to water using a CT number-to-SPR conversion table constructed for standard human tissues [15]. In charged-particle therapy for head and neck cancer, particles can traverse through the mouthpiece in some cases. As the mouthpiece is made of artificial materials, its SPR is not necessarily determined correctly from its CT number via the conversion table. Therefore, the mouthpiece may cause particle beam range errors in patients.

At QST Hospital, National Institutes for Quantum Science and Technology, in routine dose calculation of C-ion RT for head and neck cancer with a mouthpiece, the mouthpiece’s SPR is directly determined from its CT number using the calibrated CT number-to-SPR conversion table. To the best of our knowledge, dose-calculation procedures for mouthpieces have not been reported to date. In addition, the effects of the mouthpiece material on the particle beam range have not been investigated.

This study aimed to measure the SPRs of five mouthpiece materials in charged-particle therapy and investigate the SPR errors caused by the CT number-to-SPR conversion. Moreover, to evaluate the SPR error of a mouthpiece made of an ethylene–vinyl acetate copolymer (EVA) on patient dose distribution, we compared the dose distribution calculated from the SPR of the mouthpiece using the CT number-to-SPR conversion table with the recalculated dose distribution using the SPR obtained from the present study.

## Materials and methods

### Mouthpiece materials used

We investigated five mouthpiece materials used in charged-particle therapy for head and neck cancer to evaluate the SPR error and its impact on the dose distribution. The characteristics of the materials used in this study are summarized in Table 1. We routinely use the Nipoflex 710™ (NIP) (Tosoh Corporation, Tokyo, Japan), an EVA resin. The NIP and the Bioplast™ clear soft plate (BIO) (SCHEU-DENTAL GmbH, Iserlohn, Germany) are thermoplastic EVA resins used in dentistry for the treatment of temporomandibular disorders and as protective mouth guards in contact sports. The Exafine Putty Type™ (EXA) (GC Corporation, Tokyo, Japan) is a vinyl polysiloxane that is used as an impression material for making dentures. Clear photoreactive resin for Formlabs 3D printers (3DP) (Formlabs Inc., MA, USA) is a light-cured resin that is used to make 3D-printed oral stents for RT from diagnostic CT images. The Tempsmart™ (TEM) (GC Corporation, Tokyo, Japan) is a dual-cured bis-acrylic composite material that is used to create temporary crowns and bridges in dental procedures.

**Table 1:** Characteristics of the mouthpiece materials

Material	Classification of the material	Composition	Thickness (mm)	Density (g/cm^3^)
NIP	Thermoplastic resin	Ethylene–vinyl acetate copolymer	37.1 ± 0.1	0.949
BIO	Thermoplastic resin	Ethylene–vinyl acetate copolymer	35.3 ± 0	0.96
EXA	Addition curing silicone impression material	Vinyl polysiloxane, silicon dioxide, and platinum catalysts	47.4 ± 0.1	1.80
3DP	Light-cured resin	Methacrylated oligomer and methacrylated monomer	40.0 ± 0	1.09–1.12
TEM	Dual-cured bis-acrylic composite material	Silica filler and methacrylic acid ester	32.9 ± 0.1	Base: 1.2 Catalyst: 1.3

*3DP* Formlabs 3D printers, *BIO* Bioplast™ clear soft plate, *EXA* Exafine Putty Type™, *NIP* Nipoflex 710™, *TEM* Tempsmart™

### Measurement of stopping-power ratio

The SPRs of the mouthpiece materials were measured using 292.3 MeV/u monoenergetic carbon beams at QST Hospital. One-mm-wide ripple filters made of polymethyl-methacrylate and aluminum were used to mitigate the effect of range straggling due to the intervening mouthpiece’s material. The SPRs of the NIP and BIO were measured at the fixed port, whereas the SPRs of the EXA, 3DP, and TEM were measured at the rotating gantry port. An in-house parallel plate ionization chamber with a 150-mm-diameter circular sensitive area was inserted into a motor-driven water tank, and a mouthpiece material was introduced at its upstream surface. The integral depth dose (IDD) of the C-ion beam was measured with and without the mouthpiece material in place.

The SPR (*ρ*_s_) of the material was determined from the change in the water equivalent path length of the C-ion beam due to insertion of the mouthpiece material. The SPR represents the ratio of *t*_w_ (water equivalent thickness of the mouthpiece material) and the geometrical thickness (*t*_g_) of the mouthpiece material expressed by the following formula:

1 $$\rho_{{\text{s}}} = \, t_{{\text{w}}} /t_{{\text{g}}} ,$$

where *t*_g_ was measured with Vernier calipers at five locations and the thickness of the material was calculated as the average, while *t*_w_ was determined by the shift of the measured IDDs with and without the mouthpiece material using least-squares regression with spline interpolation. The uncertainty of the SPR was calculated on the assumption that the uncertainties of *t*_g_ and *t*_w_ were 0.5 mm and 0.1 mm, respectively.

### Measurement of CT numbers

Next, we measured the CT numbers of the five mouthpiece materials. To adapt the effect of beam hardening on CT imaging of actual head and neck cancer cases, CT imaging was performed in a water-filled cylindrical container with an outer diameter of 20 cm (inner diameter, 18 cm). All CT images were obtained using an Aquilion ONE CT scanner (Canon Medical Systems Corporation, Otawara, Japan). CT imaging parameters were matched to those usually used for head and neck planning (tube voltage, 120 kV; tube current, 50 mA; field of view, 500 mm; reconstruction kernels; and adaptive iterative dose reduction three-dimensional with a single-energy metal artifact reduction algorithm).

The CT images were imported to the RT planning support software (MIM software™ ver 6.8.4; MIM Software Inc., Cleveland, OH, USA), and the mouthpiece material in the CT image was contoured as the region of interest (ROI). The CT number of the material was determined as the average CT number in the ROI. The error in CT numbers was calculated from the standard deviation of the CT numbers in the mouthpiece.

### CT number-to-SPR conversion table

A CT number-to-SPR conversion table was constructed for the CT scanner based on the stoichiometric calibration method developed by Kanematsu et al. [15], in which 11 International Commission on Radiological Protection-determined body tissues were assumed to be representative of the human body [16]. The conversion method was validated elsewhere [17] and has been clinically used in our institution.

### SPR errors

The SPRs of the mouthpiece materials were measured using a C-ion beam. Independently, the SPRs of the materials were predicted from their CT numbers via the CT number-to-SPR conversion table. The SPR errors were subsequently determined as the difference between the predicted and measured SPR values.

### Clinical evaluation

We evaluated the effect of mouthpiece-induced range error on dose distribution in a single clinical case of head and neck cancer in a patients treated previously with C-ion RT at QST Hospital using the NIP mouthpiece. We selected a case of hard palate mucosal malignant melanoma treated with C-ion RT of four beams passing through thick mouthpiece materials. The selected patient was edentulous in the maxilla, and the mouthpiece was made to cover the maxillary mucosa similar to a complete maxillary denture. The dimensions of the mouthpiece were 40 mm (depth), 60 mm (width), and 25 mm (height), and the maximum distance that the C-ion beam could pass through was 60 mm. In C-ion RT for oral mucosal melanoma, gross tumor volume (GTV) including melanosis was defined as the gross extent of the tumor based on intraoral examination, CT imaging, and magnetic resonance (MR) imaging findings. The clinical target volume (CTV) was defined as the GTV with a margin of 5–10 mm. The planning target volume (PTV) was determined by adding a margin of 2–3 mm to the CTV.

In the patient dose calculation of C-ion RT, the SPR of the mouthpiece material was not overwritten by the measured or nominal values; instead, it was directly derived from the CT number using a calibrated conversion table. The planned dose distribution was recalculated by overwriting the SPR of the mouthpiece using the measured SPR of the NIP. The recalculated dose distribution was compared with the corresponding planned dose distribution and analyzed using the differential dose distribution and dose-volume histograms (DVHs). The original treatment plan and patient dose recalculation were made using the Xio-N™ treatment planning system (ELEKTA, Stockholm, Sweden; Mitsubishi Electric, Tokyo, Japan). All patients provided informed consent authorizing the use of their personal information for research purposes. This study was reviewed and approved by the Institutional Ethical Committee on Human Clinical Research (20-040) and conducted in accordance with the Declaration of Helsinki.

## Results

**Fig. 1:**
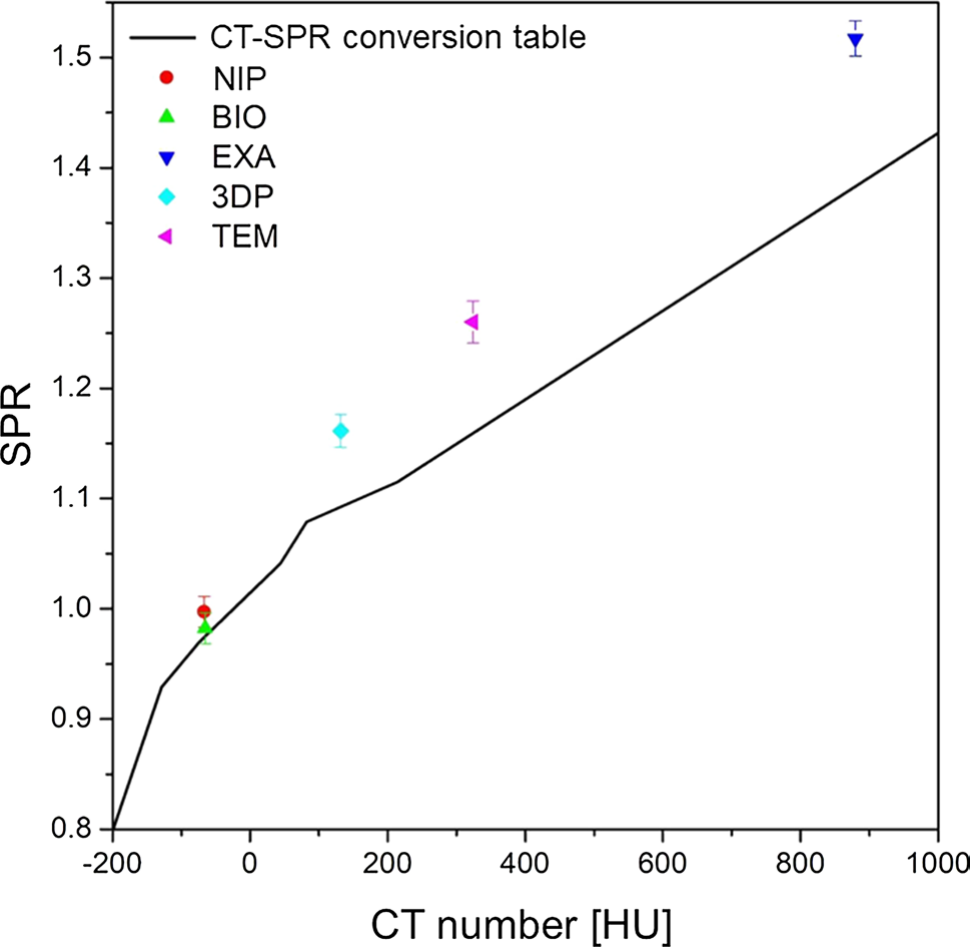
Computed tomography number-to-SPR conversion table (solid line) and SPR for each of five mouthpiece materials. SPR, stopping-power ratio

The IDDs measured with and without the mouthpiece were compared to obtain the water equivalent thickness of each material (*t*_w_). CT numbers, SPRs measured with *t*_w_ and *t*_g_, SPRs predicted from the CT numbers using the CT number-to-SPR conversion table, and the SPR errors for the five measured materials are shown in Table 2. Additionally, the SPRs of the five materials were plotted on the CT number-to-SPR conversion table to evaluate their tissue equivalencies (Fig. 1).

**Table 2:** CT numbers and stopping-power ratios (SPRs) of each material

Material	CT number (HU)	Measured SPR	Predicted SPR	Error
NIP	− 66.85 ± 11.91	0.997 ± 0.014	0.974	0.023
BIO	− 65.45 ± 10.35	0.982 ± 0.014	0.975	0.007
EXA	879.79 ± 31.16	1.517 ± 0.016	1.383	0.134
3DP	131.94 ± 11.82	1.161 ± 0.015	1.093	0.068
TEM	324.15 ± 17.46	1.260 ± 0.019	1.159	0.101

*3DP* Formlabs 3D printers, *BIO* Bioplast™ clear soft plate, *CT* computed tomography, *EXA* Exafine Putty Type™, *HU* Hounsfield unit, *NIP* Nipoflex 710™, *TEM* Tempsmart™

**Fig. 2:**
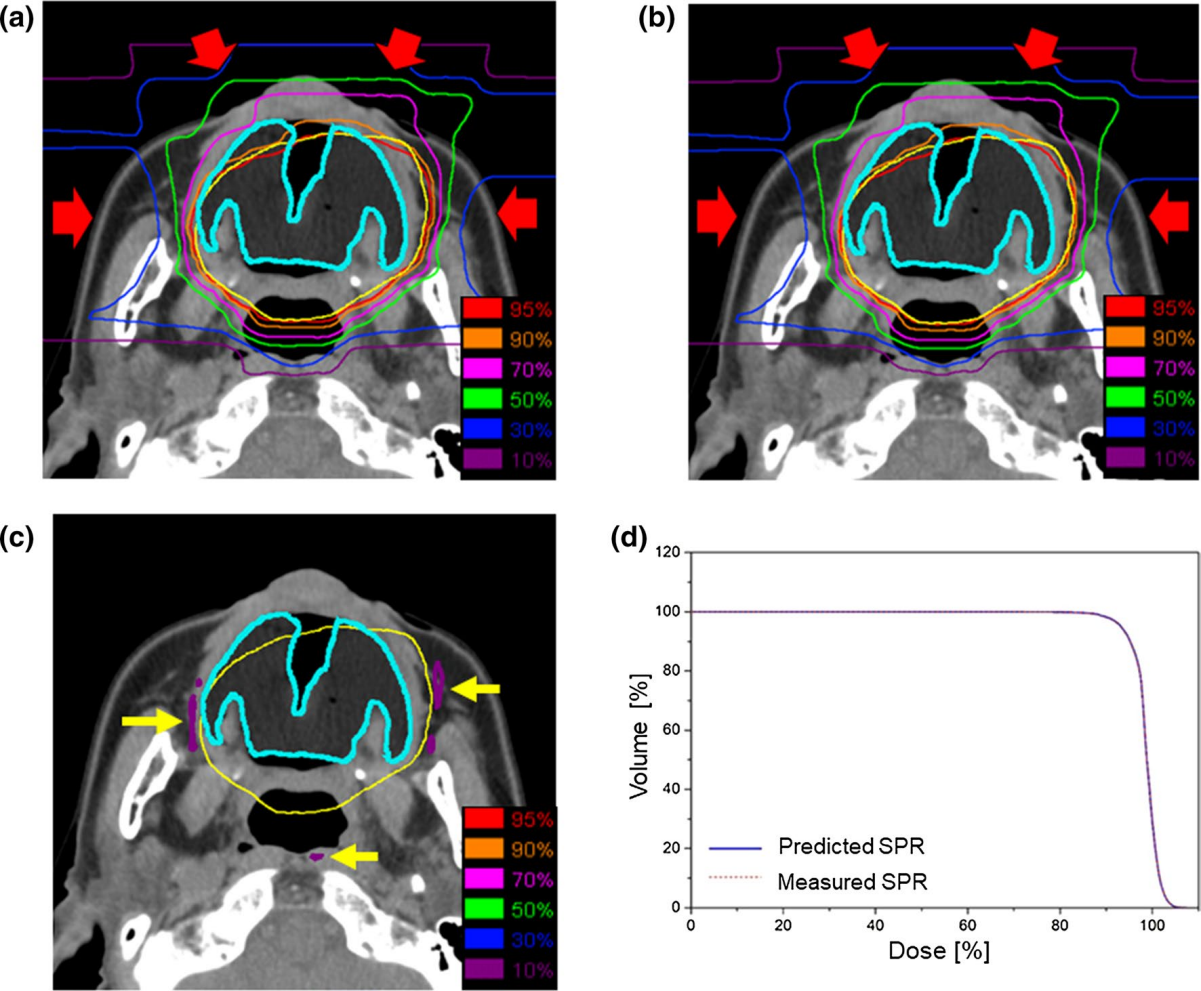
Axial computed tomography (CT) images with dose distribution and dose-volume histogram in head and neck cases irradiated by a passive irradiation method with a beam passed through the mouthpiece. Carbon-ion radiation therapy was delivered at 57.6 Gy (relative biological effectiveness) using four ports. The shown isodose lines correspond to 95%, 90%, 70%, 50%, 30%, and 10% dose areas. The planning target volume and mouthpiece are demarcated by yellow and cyan lines, respectively. **a** Dose distribution from predicted stopping-power ratio (SPR) calculated from the CT numbers of the mouthpiece using the CT number-to-SPR conversion table. **b** Dose distribution recalculated by assigning the SPR of the mouthpiece with the SPR of 1.00 obtained in the present study. **c** The differential dose distribution. There was a dose difference around the distal beam end between the planned and recalculated dose distributions (arrows). **d** Dose-volume histograms of the planning target volume. Dose-volume histogram calculated from the CT number of the mouthpiece with the SPR determined by the CT number-to-SPR conversion table (blue solid line). The dose-volume histogram was recalculated by assigning the SPR of the mouthpiece with the SPR 1.00 calculated in this study (red dotted line)

Figure 2 compares the planned dose distribution with the recalculated dose distribution determined in the present experiment by assigning the SPR of NIP as 1.00 (because the assignment function of the Xio-N™ limits the resolution of the SPR to two decimal places, 0.997 was rounded to 1.00). The isodose line of the recalculated dose distribution was shifted by 1 mm proximal to the area where the particle beam passed through a distance of 5 cm or more of the mouthpiece. There was little difference in PTV dose coverage between the planned and recalculated dose distributions, and the DVHs of PTV in both distributions almost overlapped with each other.

## Discussion

In photon and charged-particle therapy for head and neck cancer, the effectiveness of the mouthpiece as a spacer to reduce the dose delivered to the healthy tissues has been reported [3–10, 12]. In charged-particle therapy, the beam may unavoidably pass through the mouthpiece or be stopped on the mouthpiece to spare the surrounding normal tissue. However, the CT number-to-SPR conversion table used in particle therapy has been constructed for standard human tissues [15]. Thus, it is not possible to accurately calculate the SPR of artificial materials, for example, mouthpieces, from their CT numbers using the CT number-to-SPR conversion table. Therefore, the use of a beam passing through an artificial material may result in a range error and affect treatment accuracy. Nevertheless, the SPR of the mouthpiece material has yet to be discussed. In this study, the SPR of five mouthpiece materials was measured. Materials other than EVA-based ones have low equivalence to human tissues. Among the materials evaluated, the EVA resins had a high equivalence to human tissue, with an SPR error within 2% (Fig. 1, Table 2). Therefore, the SPR of the EVA mouthpiece may be determined directly from its CT numbers using the CT number-to-SPR conversion table in charged-particle therapy treatment planning, even if the particle beam passes through the EVA mouthpiece.

To date, there have been few reports on the use of mouthpiece materials for charged-particle therapy, and the SPR of the mouthpiece material has not been reported. In C-ion RT, we previously reported the efficacy of a mouthpiece made of EVA to reduce the dose delivered to the surrounding healthy tissues in patients with head and neck cancer [3, 4]. Aponte et al. [18] reported the effectiveness of proton beam therapy at the MD Anderson Cancer Center for a combined intraoral/extraoral defect using a heat-polymerized acrylic resin. At the same cancer center, the efficacy of the light-cured resin that can be shaped with a 3DP in the hospital without a professional dentist was also reported for head and neck RT [19]. Kawamura et al. [11] reported the usefulness of vinyl polysiloxane dental impression material as a proton beam stopper to save normal tissues such as the tongue during irradiation of the oral cavity. In the present study, we found that EVAs had relatively high equivalence to human tissues, while the vinyl polysiloxane and the light-cured resin had low equivalence to human tissues, with a possible error range of 7–13% when the particle beam passed through the mouthpiece materials. The CT number-to-SPR conversion table assumes equivalency to human tissues. The values of EXA containing silicon (Si) and platinum (Pt) and TEM containing silica filler (SiO_2_) would not be close to the values of the CT number-to-SPR conversion table, because they contain metals and semi-metals that are not present in human tissues. The chemical formulation of EVA is (C_2_H_4_)_n_–(C_4_H_6_O_2_)_m_, while that of 3DP is methacrylate (C_5_H_8_O_2_). C, H, and O are the major elements of the human tissues. Therefore, the values of these materials are relatively closer to those of the CT number-to-SPR conversion table compared to those of other materials. However, their composition ratio and density differ from those of human tissues, which may have caused the discrepancy. Thus, EVA resins are among the most suitable mouthpiece materials for charged-particle therapy to achieve the recommended dose accuracy. When materials other than EVA are used for the mouthpiece, it might be better to prevent the beam from passing through the mouthpiece. Alternatively, although the number of processes may increase, it is recommended that the SPR of the mouthpiece material be overwritten by its correct value in treatment planning.

## Limitations

The present study has some limitations. First, there are various types of EVA resins that differ according to their vinyl acetate content, and the CT number-to-SPR conversion table may not be applicable to all of them. We conducted this study using two types of EVA resin. Thus, a future analysis of various types of EVA resins is required.

## Conclusion

This study showed a high tissue equivalency of the EVA mouthpieces. We could directly determine the SPR of the EVA mouthpiece from its CT number using the CT-number-to-SPR conversion table, even if the particle beam passed through the EVA mouthpiece. Our results suggested that EVA is a suitable mouthpiece material for use in charged-particle therapy.
